# Partial intrauterine device migration into the abdominal cavity with bladder penetration and stone formation: A case report and multidisciplinary management

**DOI:** 10.1097/MD.0000000000045184

**Published:** 2025-10-10

**Authors:** Wenhua Liu, Yue Zhang, Liang Qian, Zhiyong Zhang

**Affiliations:** a Department of Obstetrics and Gynecology, Hangzhou Women’s Hospital, Hangzhou, China; b Department of Urology, Hangzhou Women’s Hospital, Hangzhou, China.

**Keywords:** bladder perforation, interdisciplinary management, intrauterine device migration, minimally invasive surgery, vesical calculus

## Abstract

**Rationale:**

Partial intrauterine device (IUD) migration into the abdominal cavity with bladder penetration and secondary calculus formation represents a rare complication. This case underscores the critical role of multimodal imaging and interdisciplinary collaboration.

**Patient concerns:**

A 30-year-old woman presented with refractory urinary symptoms and recurrent infections a decade after the insertion of a copper “Mother” IUD (a common type of copper IUD in China). Initial ultrasonography suggested intrauterine metallic echoes. Hysteroscopy retrieved metallic fragments but failed to locate the intact device, necessitating further investigation.

**Diagnoses:**

Abdominopelvic computed tomography revealed a migrated intrauterine device within the bladder with a 1.0 cm calculus, with 1 arm having eroded through the anterior bladder wall and a portion of the device located within the abdominal cavity.

**Interventions:**

A combined endoscopic approach was performed: cystoscopic extraction of the vesical segment of the migrated IUD arm along with its adherent calculus, followed by laparoscopic repair of the 5-mm bladder defect.

**Outcomes:**

The patient had an uneventful postoperative recovery with complete resolution of symptoms and no recurrence of infection or lower urinary tract symptoms at the 6-month follow-up.

**Lessons:**

In patients with suggestive symptoms, cross-sectional imaging should be considered early. Failed hysteroscopic retrieval definitively warrants computed tomography imaging to exclude complex ectopic migration. Multidisciplinary coordination is imperative for managing multicompartment migrations.

## 1. Introduction

Intrauterine devices (IUDs), utilized by approximately 14% of reproductive-age women worldwide, are highly effective contraceptives.^[1]^ Although generally safe, uterine perforation and device migration occur in 0.1% to 2% of cases, potentially causing visceral injury and secondary lithogenesis.^[1]^ The most common sites of ectopic migration include the omentum, bowel, and bladder. Standard diagnostic evaluation typically involves ultrasonography, with plain radiography used to confirm device presence if strings are not visible. Hysteroscopy is the primary method for retrieving devices suspected to be embedded within the uterine cavity. Partial migration into the abdominal cavity with concomitant bladder penetration is exceptionally rare and diagnostically challenging due to nonspecific symptoms such as recurrent cystitis.^[2]^ While several case reports describe complete IUD migration into the bladder or abdominal cavity, cases of partial migration with simultaneous involvement of the abdominal cavity and bladder, complicated by stone formation, are exceedingly rare.^[1–3]^ We report a case of partial “Mother” IUD migration into the abdominal cavity with bladder penetration and stone formation, highlighting interdisciplinary management strategies.

## 2. Case report

A 30-year-old gravida 1 para 1 woman underwent copper “Mother” IUD insertion 10 years previously, following an uncomplicated vaginal delivery 6 months prior. She had no history of other uterine surgeries or interventions. One year post-insertion, she developed recurrent urinary tract infections (UTIs), characterized by dysuria, frequency, and occasional suprapubic pain, occurring 3 to 4 times annually. These episodes were empirically managed with oral antibiotics (e.g., ciprofloxacin) by primary care physicians, with temporary symptom relief. Urine cultures, when performed, typically showed *Escherichia coli* growth. Due to persistent symptoms and new plans for conception, she sought IUD removal at our institution. At her most recent presentation, she reported ongoing urinary frequency and dysuria but denied hematuria, fever, flank pain, or septic features. Physical examination revealed no abdominal tenderness or suprapubic mass. Pelvic examination showed no abnormal vaginal discharge, and the IUD strings were not visible at the external cervical os. Urinalysis revealed pyuria but no hematuria.

## 3. Diagnostic evaluation

**Figure 1.**
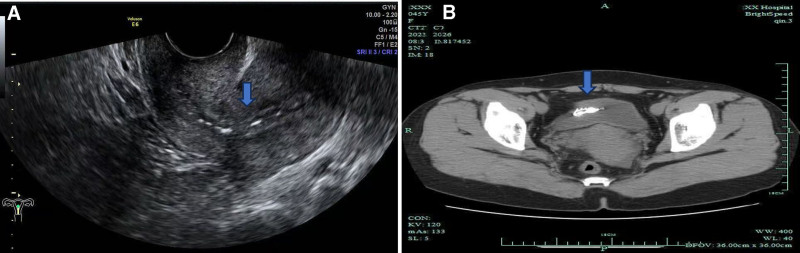
(A) Transvaginal ultrasound: intrauterine metallic focus. (B) Axial CT: abdominal IUD segment (blue arrow). CT = computed tomography, IUD = intrauterine device.

Initial evaluation included pelvic examination and transvaginal ultrasonography. The pelvic exam revealed no visible IUD strings. Transvaginal ultrasound subsequently identified a hyperechoic intrauterine metallic focus (Fig. 1A). Given the absent strings and suggestive history, hysteroscopy was performed under anesthesia the following day. Several metallic fragments were retrieved; however, the intact IUD was not visualized. Due to the failed retrieval and high suspicion of migration, abdominopelvic computed tomography (CT) was performed on the third day. CT imaging confirmed the diagnosis, revealing: a 1.0 cm calculus (radiodensity approximately 800 Hounsfield units) attached to the intravesical section of 1 arm; another arm penetrating the anterior bladder wall; the main body of the IUD located within the bladder cavity (Fig. 1B). No distant migration or intraperitoneal leakage was evident. A multidisciplinary consultation involving gynecology and urology teams was held immediately to plan further management.

## 4. Surgical management

**Figure 2.**
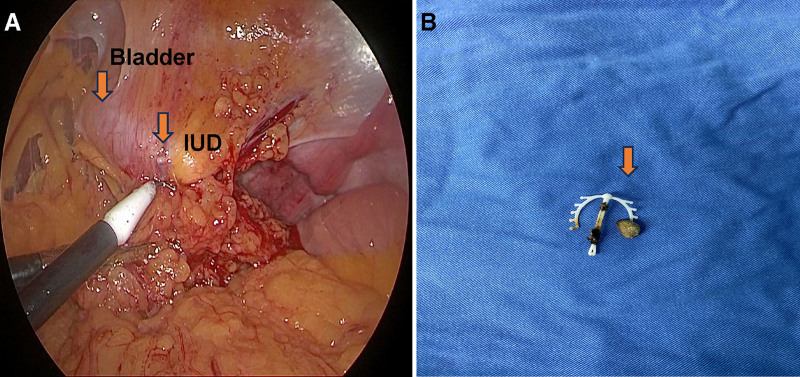
(A) Laparoscopic view of bladder repair. (B) Retrieved IUD with adherent calculus (yellow arrow). IUD = intrauterine device.

On the fourth day, a combined endoscopic procedure was performed under general anesthesia with prophylactic broad-spectrum antibiotics. A 26F continuous-flow cystoscope with a grasping forceps was used to extract the calculus intact and IUD under direct vision transurethrally. Laparoscopy was then performed to repair the 5-mm vesical perforation at the penetration site using interrupted 3 to 0 absorbable sutures (Fig. 2A, B).

## 5. Postoperative course

A transurethral catheter was maintained for 5 days after the surgery. The catheter was removed after a voiding trial confirmed the patient’s ability to empty her bladder without discomfort or significant residual volume. The patient resumed normal voiding immediately without complications.

## 6. Outcomes

At the 6-month follow-up, the patient reported complete symptom resolution with no complications and was actively pursuing conception.

## 7. Discussion

This case exemplifies the diagnostic and therapeutic challenges posed by a rare, complex migration pattern of an IUD-partial displacement into the abdominal cavity with bladder penetration and secondary stone formation. It highlights the limitations of initial diagnostic modalities and underscores the critical need for interdisciplinary collaboration when retrieval fails.

The onset of recurrent UTIs 1 year post-insertion suggests that bladder penetration likely occurred early. The process of transmural migration through the bladder wall and subsequent formation of a 1.0 cm calculus represents a long-term complication that evolved over years. The copper components likely incited a persistent inflammatory response, facilitating transmural migration.^[3]^ The long-term interaction between the intravesical copper arm and urine served as a nidus for mineralization, resulting in a calculus composed primarily of calcium salts – a recognized consequence of urinary foreign bodies.^[4,5]^

This case underscores significant diagnostic limitations. The vesical calculus was missed on initial ultrasonography, likely due to acoustic shadowing from intrauterine metallic fragments and the limited field of view of transvaginal ultrasound for evaluating the bladder dome.^[6]^ While hysteroscopic retrieval of intrauterine fragments provided false reassurance, it failed to reveal the migrated main body and perforating arm. This demonstrates that hysteroscopy alone is insufficient to rule out complex ectopic migration when the entire device is not accounted for.^[3,7]^

The crucial role of cross-sectional imaging was evident. Abdominopelvic CT, with its superior spatial resolution and multiplanar reconstruction capabilities, was pivotal in establishing the definitive diagnosis. CT precisely localized the main IUD body within the bladder lumen, with 1 arm penetrating the bladder wall, thereby accurately defining the complex anatomy and guiding the surgical strategy. This case suggests that in IUD users with refractory urinary symptoms, even if intrauterine echoes are seen, a low threshold for cross-sectional imaging before or concurrently with planned hysteroscopy may be prudent to avoid unnecessary procedures and guide definitive surgical planning.^[3,8]^

While bladder penetration is among the most common sites for complicated IUD migration,^[9]^ our case demonstrates the rarer phenomenon of partial migration where the device straddled multiple anatomical compartments (uterine cavity, abdominal cavity, and bladder). This differs from cases of complete migration to a single site and necessitates a more complex diagnostic and therapeutic approach. The successful outcome was predicated on a coordinated, minimally invasive strategy involving both gynecology and urology. The combined use of hysteroscopy, cystoscopy, and laparoscopy minimized morbidity. Cystoscopic extraction successfully removed the perforating arm and the adherent calculus without open cystotomy. Laparoscopy allowed safe and precise repair of the bladder perforation. This multi-portal endoscopic approach represents the optimal strategy for such complex migrations.^[8]^

Preoperative planning and joint execution by gynecological and urological teams were essential for comprehensive management, addressing the uterine cavity (fragments), the bladder (foreign body, stone, perforation), and the abdominal cavity efficiently and safely in a single operation. The decade-long delay between symptom onset and definitive diagnosis highlights a critical concern. Persistent urinary symptoms in IUD users, especially those resistant to standard treatment, must prompt expedited and thorough investigation for potential migration to prevent complications like stone formation, organ perforation, and fistula development.^[10,11]^

Based on this experience, we recommend a heightened index of suspicion for IUD migration in users presenting with persistent pelvic or urinary symptoms such as recurrent UTIs, dysuria, hematuria, urinary frequency, or pelvic pain, regardless of initial evaluation results or time since insertion.^[3,12]^ Furthermore, we strongly advocate for prompt cross-sectional imaging after failed retrieval; if hysteroscopic retrieval fails to locate the intact IUD or only fragments are found, abdominopelvic CT should be performed immediately for definitive localization.^[3,8]^ Moreover, cases involving confirmed extrauterine migration, particularly complex migrations involving multiple compartments, require preoperative planning and intervention by a combined gynecologic and urologic team.^[8,10]^ Finally, patient education is crucial; IUD users should be counseled during insertion and at follow-up visits to report persistent or unusual pelvic/urinary symptoms promptly, even years after placement.^[3,12,13]^

## 8. Conclusions

Partial migration of an IUD into the abdominal cavity with bladder penetration and secondary stone formation is an exceptionally rare but serious complication. It demands a high index of suspicion in symptomatic patients, even years after insertion. Overreliance on initial ultrasound or hysteroscopy can lead to significant diagnostic delays. Failed hysteroscopic retrieval is a strong indicator of potential complex migration and mandates prompt cross-sectional imaging CT. Abdominopelvic CT is the gold standard for defining the precise location and associated complications. Successful management of such complex migrations hinges entirely on a collaborative, minimally invasive approach between gynecology and urology. Persistent urinary symptoms in IUD users warrant a thorough evaluation specifically designed to exclude device migration.

## Acknowledgments

We thank the Department of Radiology at Hangzhou Women’s Hospital for their expert imaging interpretation and the urology operating team for their skilled collaboration. We also extend our gratitude to the patient for providing informed consent for the publication of this case.

## Author contributions

**Funding acquisition:** Yue Zhang.

**Investigation:** Liang Qian, Zhiyong Zhang.

**Methodology:** Wenhua Liu, Liang Qian, Zhiyong Zhang.

**Project administration:** Yue Zhang.

**Writing – original draft:** Wenhua Liu.

**Writing – review & editing:** Yue Zhang.
